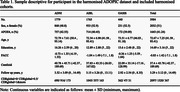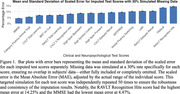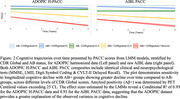# Harmonization of data from neuropsychological tests used in different prospective studies‐ Descending a Tower of Babel

**DOI:** 10.1002/alz.085009

**Published:** 2025-01-03

**Authors:** Rosita Shishegar, Timothy Cox, Shaun J Markovic, Bhargav Tallapragada, Pierrick Bourgeat, Vincent Dore, Simon M Laws, Tenielle Porter, Samantha C. Burnham, Azadeh Feizpour, Michael S. W. Weiner, Jason J. Hassenstab, John C. Morris, Christopher C. Rowe, Victor L Villemagne, Colin L. Masters, Jurgen Fripp, Yen Ying Lim, James D. Doecke, Hamid R. Sohrabi, Paul Maruff

**Affiliations:** ^1^ Austin Health, Heidelberg, VIC Australia; ^2^ School of Psychological Sciences and Turner Institute for Brain and Mental Health, Monash, VIC Australia; ^3^ The Australian e‐Health Research Centre, CSIRO, Parkville, VIC Australia; ^4^ The Australian e‐Health Research Centre, CSIRO, Canberra, ACT Australia; ^5^ Murdoch University, Murdoch Australia; ^6^ Centre for Healthy Ageing, Murdoch University, Perth Australia; ^7^ Murdoch University, Perth, Western Australia Australia; ^8^ CSIRO, Brisbane, QLD Australia; ^9^ The Australian e‐Health Research Centre, Commonwealth Scientific and Industrial Research Organisation, Brisbane, QLD Australia; ^10^ Australian E‐Health Research Centre, CSIRO, Melbourne, VIC Australia; ^11^ CSIRO Health and Biosecurity, Australian E‐Health Research Centre, Brisbane, QLD Australia; ^12^ Department of Molecular Imaging, Austin Health, Melbourne, VIC Australia; ^13^ Centre for Precision Health, Edith Cowan University, Joondalup, Western Australia Australia; ^14^ Eli Lilly and Company, Indianapolis, IN USA; ^15^ Austin Health, Melbourne, VIC Australia; ^16^ Department of Molecular Imaging & Therapy, Austin Health, Melbourne, VIC Australia; ^17^ The Florey Institute of Neuroscience and Mental Health, Melbourne, VIC Australia; ^18^ University of California, San Francisco, San Francisco, CA USA; ^19^ Washington University in St. Louis, St. Louis, MO USA; ^20^ Washington University in St. Louis, School of Medicine, St. Louis, MO USA; ^21^ Molecular Research and Therapy, Austin Health and University of Melbourne, Heidelberg, VIC Australia; ^22^ Departments of Medicine and Molecular Imaging, University of Melbourne, Austin Health, Melbourne, VIC Australia; ^23^ The Florey Institute of Neuroscience and Mental Health, The University of Melbourne, Parkville, VIC Australia; ^24^ Department of Molecular Imaging & Therapy, Austin Health, Heidelberg, VIC Australia; ^25^ University of Pittsburgh, Pittsburgh, PA USA; ^26^ Florey Institute of Neuroscience and Mental Health, Parkville, VIC Australia; ^27^ Turner Institute for Brain and Mental Health, School of Psychological Sciences, Monash University, Melbourne, VIC Australia; ^28^ The Australian e‐Health Research Centre, CSIRO, Brisbane, QLD Australia; ^29^ Centre for Healthy Ageing, Murdoch University, Murdoch, Perth, Western Australia Australia; ^30^ Cogstate Ltd., Melbourne, VIC Australia; ^31^ Turner Institute for Brain and Mental Health, Monash University, Melbourne, VIC Australia

## Abstract

**Background:**

Cognitive dysfunction is central to clinicopathological models of Alzheimer’s disease (AD). While AD prospective studies assess similar cognitive domains, the neuropsychological tests used vary between studies, limiting potential for aggregation. We examined a machine learning (ML) data harmonisation method^1^ for neuropsychological test data to develop a harmonised PACC score for the Alzheimer’s Dementia Onset and Progression in International Cohorts (ADOPIC) consortium.

**Method:**

ADOPIC included longitudinal clinicopathological data from AIBL (N = 1765), ADNI (N = 1779) and OASIS (N = 440) cohorts (Table 1). Harmonization involved three stages. First, cognitive domains of interest and the neuropsychological tests assessing each were defined. Second, a standardized scoring and naming convention was established for demographic and neuropsychological outcomes. Third, the ML harmonisation approach^1^ was applied. Test scores present in one cohort, but not another, were treated as missing and imputed using ML before calculating a PACC^1^. Imputation utilized data from neuropsychological tests, age, sex, years of education, and *APOE*‐ɛ4 status. We validated the harmonized PACC (H‐PACC) by randomly simulating missing cognitive scores and analysing percentage error of imputed scores versus actual data^1^. Validity of harmonized scores was determined by their sensitivity to decline associated with amyloid positivity and clinical disease stage. Linear mixed models (LMMs) modelled trajectory of change on H‐PACC and AIBL PACC scores (including identical tests), with time, CDR‐global score, and amyloid status (Aβ‐/Aβ+) interactions as fixed effects. Sex and age at baseline were covariates. Variation in baseline and decline in PACC scores was modelled using random intercepts and slopes. Effect sizes for LMMs were computed with pseudo‐R‐squared.

**Result:**

Percentage errors for imputed neuropsychological test scores showed high accuracy with low standard deviations (Figure 1). Sensitivity to clinicopathological disease stage was qualitatively similar for the H‐PACC and AIBL PACC (Figure 2), both discriminating (p<0.001) annual decline rates of Aβ+ CDR 0.5 and CDR≥1 groups from Aβ‐ CDR 0 group. Effect sizes were substantial, with H‐PACC and AIBL PACC data explaining 95% and 93% of variance, respectively.

**Conclusion:**

The H‐PACC, developed via ML harmonization, was precise and has utility for aggregating neuropsychological test data to be used in prospective AD studies.

**References**:

^1^ https://doi.org/10.1002/alz.044302

^2^ https://doi.org/10.1093/bioinformatics/btr597